# A Critical View of Specific Antibody Deficiencies

**DOI:** 10.3389/fimmu.2019.00986

**Published:** 2019-05-01

**Authors:** Ricardo U. Sorensen

**Affiliations:** ^1^Professor Emeritus of Pediatrics, Department of Pediatrics, Louisiana State University Health Science Center, New Orleans, LA, United States; ^2^Louisiana Primary Immunodeficiency Network, New Orleans, LA, United States; ^3^Honorary Professor, Faculty of Medicine, University of La Frontera, Temuco, Chile

**Keywords:** *Streptococcus pneumonia*, infections, immunity, *S. pneumoniae* vaccines, anti-polysaccharide antibodies, antibody assessment, specific antibody deficiencies (SAD), management of SAD

In thisopinion manuscript the author postulates that the present definition of Specific Antibody Deficiency (SAD) needs to be revised and expanded. It is presently defined as a syndrome of low IgG antibody responses to purified *Streptococcus pneumoniae* capsular polysaccharides vaccines with normal concentrations of IgG, IgA, IgM, and IgG subclasses. Responses to protein antigens and to pneumococcal polysaccharides conjugated to proteins are normal (1, 2). Severe, moderate and memory SAD forms have been arbitrarily defined by expert committees. These definitions miss the imperfections of a diagnosis based on mainly poorly standardized laboratory tests and curtail the search for many other clinically relevant specific deficiencies of antibodies to bacterial, viral and fungal pathogens.

## The Laboratory Tests on Which the Diagnoses of SAD are Based are Variable and Imperfect

The assessment of IgG antibodies against *S. pneumonia* serotype-specific capsular vaccine antigens are widely used to diagnose a patient's ability to develop IgG antibody responses. Available vaccines include a 23-valent purified capsular polysaccharide vaccine that includes the 23 most prevalent serotypes (PPV-23) and several polysaccharide-protein conjugated vaccines that include antibiotic resistant serotypes. These are a 7 serotypes vaccine (PCV-7) that was replaced by a 13 serotypes vaccine (PCV-13) and a different 10-valent vaccine (PCV-10). The use of both purified polysaccharide and combined protein-polysaccharide vaccines places evaluation of anti-pneumococcal immunity among the most valuable tools used to assess an important component of antibody-mediated immunity.

Different laboratory methods are used to define normal and abnormal antibody responses. The diagnosis of SAD is given to patients older than 2 years with recurrent infections who fail to mount an arbitrarily defined normal response to purified pneumococcal serotypes (3, 4). If the patient was immunized with PCV-7, PCV-13, or PCV-10, the diagnosis of SAD is only possible based on the response to serotypes not present in conjugate vaccines. A response to conjugated serotypes does not preclude unresponsiveness to pure polysaccharide antigens. Correlation of the extent of antibody deficiencies and clinical presentation is poor, the diagnostic methods used at the present time are unreliable, and having low responses as defined for SAD does not necessarily lead to recurrent infections (5).

The methods used to measure anti-pneumococcal, serotype-specific IgG antibodies that confer long-term protection are summarized in Table 1 (6, 7).

**Table 1:** Methods to measure lgG anti-S. *pneumonia* surface polysaccharide antibodies.

**Test features**	**WHO ELISA**	**Luminex^®^**	**Global test**
Performance	Cumbersome, in few, mostly research laboratories	Easier and faster. Widely used in Commercial laboratories	Easier and cheaper to perform, used in many parts of world
Serotype-specific antibodies measured	Limited, variable 12–16 serotypes	Usually to each of 23 serotypes in PPV- 23	All 23 serotypes used as one antigen
Reproducibility	High worldwide	Low among different laboratories and with WHO-ELISA	Questionable relationship to serotype-specific tests

*WHO ELISA and Luminex® are pneumococcal serotype-specific antibody assays (PSSA) with results expressed as weight /volume*.

The World Health Organization ELISA (WHO, ELISA) is well-standardized and reproducible. It defines a specific way of performing this test. It was developed under the auspices of the World Health Organization, to evaluate conjugate vaccine effectiveness. Results are expressed as μg/ml after calibration against an FDA standard now replaced by 007sp and by laboratory standards (7).

An easier to perform method that simultaneously measures antibodies to each of the 23 serotypes in the PPV-23 is based on a multiplex fluorescent bead assay, Luminex®. It is used by most reference laboratories in the United States. Luminex results are consistent within laboratories but there are significant differences among laboratories and its correlation with ELISA test results is variable (7, 8).

A “global test” measures antibodies against all 23 serotypes in the 23-valent PPV as one antigen (9). The information obtained with the global test does not correlate well with results obtained when measuring antibodies against each serotype individually.

The only functional assay of anti-pneumococcal antibodies is opsonophagocytosis (OPA). It measures antibodies of all immunoglobulin classes against polysaccharide and protein antigens on the surface of intact bacteria. This test is not generally available for clinical use although vaccine manufacturers use it extensively when testing vaccine antigenicity.

There is not a strict relationship between the weight by volume antibody concentration results obtained by ELISA and OPA results (10). In clinical practice, there are patients who have normal ELISA titers but who improve clinically when given therapeutic IgG, suggesting that the lower function observed in the elderly (11) may also be present in some individuals at an earlier age.

When vaccines are used to evaluate a response to *S. pneumoniae* polysaccharides, interpretation of results is based on a combination of the following: (1) increase in specific antibody concentration over pre-immunization levels, (2) the final concentration of antibodies after immunization, and (3) the percentage of serotypes to which the patient developed an arbitrarily defined antibody concentration.

There are shortcomings with each of these criteria:

1. The requirement for a minimum two- and four-fold increase disregards the fact that high pre-immunization concentrations may not increase with immunization. The desired concentration to prove effective antibody production is subject to variable interpretation. If the value is set at 1.0 instead of 1.3 μg/ml, (12) the number of responses considered normal can differ, without proof that these differences are clinically significant.
2. The relevance of the percentages of serotypes inducing a given antibody concentration is also subject to interpretation. Different combinations of high, medium, and low antibodies can be seen in the same patient sample. The serotypes that may elicit these different antibody concentrations vary from patient to patient. It is therefore not surprising that attempts to identify a response to one or several selected serotypes as representative of the response to all or most serotypes included in the PPV-23 have failed. The possibility of using serotype-specific threshold values could be applicable for some defined, uniform populations (13). However, in a diverse clinical practice this kind of definition of antibody responses is unrealistic.

The history of prior immunization with *S. pneumonia* vaccines is essential in the interpretation of antibody measurement results. Responses to conjugate polysaccharides in PCV are considered T cell-dependent while responses to purified polysaccharides in the PPV do not involve T cell activation (14–18). The response to PCV matures earlier and is developed in the first months of life while the response to PPV is considered fully developed only by 2 years of age. Prior PCV immunization induces antibodies indistinguishable from those induced by purified polysaccharides. When using PPV to assess specific antibodies to *S. pneumoniae* polysaccharides in PCV immunized individuals, antibodies to PCV serotypes do not document a normal response to PPV. The additional response to PPV may be influenced by conditioning produced be the exposure to conjugate polysaccharides (19). Conversely, a response to PPV may occur even in patients who fail to respond to PCV, documenting that conjugate and purified polysaccharides induce antibodies through different activation pathways.

The injectable polysaccharide S. typhi vaccine (Typhi Vi) has been studied as an alternative to the PPV-23 to assess the polysaccharide response. Typhi Vi antibodies are usually absent in the population. Therefore, they reflect a new immune response but their results are not concordant with results of anti *S. Pneumonia* polysaccharides (20–22). Like other vaccines, the typhi does not test the same molecular pathways activated by *S. pneumonia* capsular polysaccharides.

## There are Different Forms of Specific Antibody Deficiencies

An experience-based list of abnormalities in the response to various forms of exposure to pneumococcal polysaccharides is presented below. Correctly identifying these patterns of anti-*S. pneumonia* polysaccharide responses in patients with increased susceptibility to infections allows the clinician to define patient-based management options.

The response patterns to conjugate and purified polysaccharide vaccines that suggest an immune dysfunction are as follows:

1. Deficient response to pure polysaccharides (SAD or PPV-SAD)
2. Deficient response to conjugate polysaccharides (PCV-SAD), frequent in children
3. Deficient response to PPV and PCV.

Options 2 and 3 are not accepted as an antibody deficiency syndrome in present disease classification

For each of the two kinds of vaccine polysaccharide antigens, the abnormality may be:

- Absent response to any serotype tested Low antibody concentration (below 1.3 μg/ml in the USA. Other cut-off values are used in different regions of the world).
- Incomplete antibody repertoire. Protective titers to < 50–80% of serotypes. This could be a strong response to only 1 serotype.
- Poor memory after adequate response to immunization.
- Deficient opsonophagocytosis. Serological response may appear normal, but antibodies are not protective.

^*^All these situations may be the only detected immune abnormality or part of other immune abnormalities. They may also be present in children and adults who do not experience any form of severe or recurrent infections.

The multiplicity of immunological phenotypes and conditions in which a specific antibody deficiency can be observed suggests that different pathogenic mechanisms cause this defect. A lack of response is different from a deficient immunological memory capable of maintaining protective IgG concentrations. Antibodies against purified or conjugate polysaccharides do develop through different cellular pathways likely to be affected by different abnormalities (23, 24). Additionally, the biologic diversity of serotype-specific polysaccharides leads to very specific lack of responses to some polysaccharides in some individuals. The lack of responses to *S. pneumonia* polysaccharides may be linked to unresponsiveness to unrelated antigens as well. In some young patients the selective antibody deficiency may just represent an extension of an immunological status that is normal in infants (25).

Further variability in the pathogenesis of anti-polysaccharide antibody deficiencies is suggested by the large number of more general immunodeficiencies, such as IgG subclass deficiencies (26–30), and by the wide array of known immunodeficiency syndromes that may have normal IgG concentrations, and poor or absent polysaccharide responses. Examples are patients with asplenia, hyper-IgE syndrome, or selective IgA deficiency (31–36). Additionally, congenital molecular abnormalities like Bruton's tyrosine kinase deficiency, commonly associated with agammaglobulinemia, may have SAD (37). Similar unique associations between molecular abnormalities and deficient specific antibody responses are increasingly identified as evaluation of anti-*S. pneumoniae* antibodies has become part of the evaluation of patients with different forms of immunodeficiencies (38, 39).

## Vaccines are Important Treatment Elements

The management of abnormalities of the response to pneumococcal polysaccharides includes, foremost, an adequate identification of infections, infection complications and their impact on cost and quality of life. Based on the type of abnormality detected it is possible to use additional immunization, preventive antibiotics and, in some patients, the use of IgG replacement.

*S. pneumoniae* vaccines are a cost-effective part of treatment. Below are the recommended additional immunizations of patients with an identified abnormal antibody response to pneumococcal polysaccharides:

PPV non-responders (classic SAD) PCV 13 (29)

PCV-non-responders PPV (40)

Normally, unimmunized older children and adults have develop antibodies to most *S. pneumonia* serotypes in response to clinical and subclinical infections. Such unimmunized patients usually respond serologically and clinical to immunization with PCV-13 followed by PPV-23.

The use of PPV-23 in patients unresponsive to a full complement of conjugate vaccines is one of the most useful and cost effective form of treatment of this frequently observed condition. Estrada el al. observed that the PPV-23 vaccine serologically and clinically improved children who had failed to develop strong antibody responses and had recurrent infections despite a full complement of PCV vaccines (40). A general stimulating effect of immunity was also reported (41). Notably, it did not matter if the infections were caused by pneumococci, other bacterial pathogens, or respiratory viruses. Although the PPV-23 vaccine is recommended only after the second year of life, Tang et al. observed strong responses in unimmunized 12 month-olds (42). Personal observation confirms strong antibody responses to the PPV-23 in patients between 1 and 2 years of age. While not recommending this course of action, considering the large difference in cost of one dose of PPV-23 as opposed to several doses of the PCV-10 and PCV-13, in economically strapped situations or areas in which PCV is not available, the earlier use of PPV-23 may be advisable.

## Treatment Trials With Human Gammaglobulin Should be Considered in Special Clinical Circumstances

In patients with recurrent infections and laboratory diagnosed forms of Specific Antibody Deficiencies, IgG replacement is an accepted treatment option. This could be used for a period of time in young children and probably for life in SAD forms detected in adolescents and adults.

It is also of note that patients without a clear immunoglobulin-deficiency syndrome or immunologically mild forms of SAD are very likely to experience a very significant reduction of infections and improvement in quality of life if they are treated with IgG replacement. IgG replacement should be based on a complete assessment of the patient's condition and not only on the presence or absence of anti-pneumococcal antibodies (43). A therapeutic trial of 6–12 months of treatment with subcutaneous or intravenous IgG with a rigorous assessment of infections and quality of life is an option strongly recommended by this author. It is important, however, to use all available treatment approaches before resorting to IgG replacement.

In summary, our present diagnosis and treatment of specific antibody deficiencies need reevaluation and improvement. The most reliable evidence of a failure of antibody-mediated immunity is the continued proven presence of infections that improve with intensified treatment. Treatment can be with antibiotics in the case of bacterial infections or with human gammaglobulin when antibiotics fail or the infections are caused by viruses. Given the complexity of assessing true antibody function, rather than further attempts to define numeric criteria for the normality or deficiency of antibodies, it is important to develop a better definition of infections that signal a deficient antibody function. Acceptable criteria for therapeutic trials of IgG replacement of sufficient length and duration, and objective criteria to assess the patient's clinical response and the cost effectiveness of treatment need to be developed with the aim of helping patients and preventing the abuse of treatment with IgG.

## Author Contributions

The author confirms being the sole contributor of this work and has approved it for publication.

### Conflict of Interest Statement

The author declares that the research was conducted in the absence of any commercial or financial relationships that could be construed as a potential conflict of interest.
